# gp91^phox^, a Novel Biomarker Evaluating Oxidative Stress, Is Elevated in Subclinical Hypothyroidism

**DOI:** 10.1155/2020/3161730

**Published:** 2020-05-06

**Authors:** Xiaochun Ma, Furong Wang, Xiaowen Zhen, Lifang Zhao, Li Fang, Zhenfang Dong, Wenbin Chen, Xiaoming Zhou

**Affiliations:** ^1^Department of Cardiovascular Surgery, Shandong Provincial Hospital Affiliated to Shandong First Medical University, Jinan, 250021 Shandong, China; ^2^Department of Pharmacology, College of TCM, Shandong University of Traditional Chinese Medicine, Jinan, 250355 Shandong, China; ^3^Department of Endocrinology, Shandong Provincial Hospital Affiliated to Shandong First Medical University, Jinan, 250021 Shandong, China; ^4^Shandong Clinical Medical Center of Endocrinology and Metabolism, Jinan, 250021 Shandong, China; ^5^Institute of Endocrinology and Metabolism, Shandong Academy of Clinical Medicine, Jinan, 250021 Shandong, China; ^6^Department of Clinical Laboratory, Shandong Provincial Hospital Affiliated to Shandong First Medical University, Jinan, 250021 Shandong, China; ^7^Scientific Center, Shandong Provincial Hospital Affiliated to Shandong First Medical University, Jinan, 250021 Shandong, China

## Abstract

**Background:**

gp91^phox^, the catalytic core of NADPH oxidase (NOX) and biomarker of NOX activation, has been recently recognized as a parameter of systemic oxidative stress in several studies. Subclinical hypothyroidism (SH) is characteristic of elevated level of serum thyroid stimulating hormone (TSH) and is frequently accompanied with cholesterolemia. In this study, the levels of serum soluble gp91^phox^ were measured to assess the oxidative stress in patients with SH. And the relationship among gp91^phox^, low-density lipoprotein-C (LDL-C), and TSH was also investigated.

**Methods:**

A total of 51 subjects were enrolled and categorized into four groups: the healthy controls subjects (*n* = 13), controls with high level of LDL-C alone (*n* = 12), SH with normal level of LDL-C (*n* = 11), and SH with high level of LDL-C (*n* = 15). The related clinical and laboratory data were collected for statistical analysis. All the patients were newly diagnosed and did not take any medication. The information of lipid profile and thyroid function was extracted, and the concentrations of gp91phox were obtained with ELISA.

**Results:**

The levels of serum soluble gp91phox evidently increased in the patients with SH with a high level of LDL-C (81.52 ± 37.00 ug/mL) as compared to the healthy controls (54.98 ± 1.83ug/mL, *p* < 0.001), controls with high level of LDL-C (61.21 ± 4.48 ug/mL, *p*=0.038) and SH with a normal level of LDL-C (62.82 ± 11.67ug/mL, *p*=0.027). Additionally, the levels of gp91^phox^ showed a significant positive correlation with both the levels of LDL-C (*r* = 0.595, *p* < 0.001) and TSH (*r* = 0.346, *p*=0.013) by the Spearman correlation analyses. The correlation remained significant even when the effect of another factor was controlled (TSH: when the effect of LDL-C was controlled, *r* = 0.453, *p*=0.001; LDL-C: when the effect of TSH was controlled, *r* = 0.291, *p*=0.040). The main effect analysis showed an independent main effect of either LDL-C (*p* = 0.041) or TSH (*p*=0.022) on gp91^phox^ without interaction (*p*=0.299).

**Conclusions:**

Our work demonstrated that the levels of gp91^phox^, a novel biomarker for measuring the oxidative stress, were significantly elevated in the patients with SH. And LDL-C and TSH were both independent predictors of gp91^phox^. *Abbreviations.* BMI : Body mass index; TC : Total cholesterol; LDL-C : Low-density lipoprotein cholesterol; HDL-C : High-density lipoprotein cholesterol; TG : Triglyceride; FBG : Fasting blood glucose; FT3 : Free triiodothyronine; FT4 : Free thyroxine; TSH: Thyroid stimulating hormone; SBP : Systolic blood pressure; DBP : Diastolic blood pressure; SD : Standard deviation; LSD: Least significant difference.

## 1. Introduction

Subclinical hypothyroidism (SH) is referred to as the condition where the levels of serum thyroid stimulating hormone (TSH) are elevated and the concentrations of thyroid hormones are within the reference ranges. SH occurs in 3% to 15% of general population in the whole world, and the prevalence increased markedly in adult females [1–3]. A growing number of studies have suggested that there is an increased risk of cardiovascular diseases in patients with SH [4, 5]. Those well-documented concomitant cardiovascular risk factors in SH include hypercholesterolemia, insulin resistance, impaired endothelial function, and inflammation [6–8].

Oxidative stress, an imbalance in the equilibrium favoring the increased production of free radicals and diminished antioxidants, has been demonstrated to be elevated in SH [9]. However, the results are still scarce and somewhat controversial [10–14]. It has been correlated with a wide range of biological and pathological conditions [15], especially in the cardiovascular system [16]. NADPH oxidase (NOX), one of the most important oxidases responsible for ROS formation in the cardiovascular system, plays a pivotal role in the initiation and progression of atherosclerotic diseases [17, 18]. gp91^phox^, the catalytic core of NOX and marker of NOX activation, has been recognized as a parameter of systemic oxidative stress in an array of recent studies [19, 20]. However, relevant reports are still missing on the concentrations of serum soluble gp91^phox^ in SH.

The increased intensity of oxidative stress in SH has been associated with the elevated concentrations of total cholesterol (TC) and low-density lipoprotein cholesterol (LDL-C) [21]. And several research groups have advocated that TSH could directly influence the oxidative stress in SH [22]. Thus, the extent of hyperthyrotropinemia in SH might be applied as an indicator not only of the severity of hypothyroidism but also of the extent of the associated lipid peroxidation [23]. Despite these findings, the relationship of oxidative stress with TSH and lipid profiles in SH has yet to be clarified to date.

Given the high prevalence of SH in the general population and undetermined correlation of oxidative stress with hyperthyrotropinemia and hyperlipidemia, we aimed to explore in this study the levels of gp91^phox^ as well as the effects of LDL-C and TSH on gp91^phox^ in patients with SH.

## 2. Materials and Methods

### 2.1. Study Design and Subjects

A total of 51 subjects were recruited from the Health Management Center of Shandong Provincial Hospital. All patients were newly diagnosed. To avoid the confounding effects on the oxidative stress, those subjects were excluded based on the criteria as follows: (1) with any sign of acute infection; (2) with chronic infections including chronic gastroenteritis, nephritis, bronchitis, nephritic syndrome, or autoimmune hepatitis; (3) having a history of hypertension, diabetes mellitus, coronary heart disease, malignant cancers, or cardiac/renal/hepatic/thyroid dysfunction; (4) administrated with drugs such as lipid-lowering drugs, antioxidant vitamin supplements, or hormone replacement therapy; (5) smoker, alcoholics, long-time tea drinking, and having recent heavy physical. The study was approved by the Ethics Committee of Shandong Provincial Hospital. Written informed consent was obtained from each subject prior to their enrollment to the study.

As the level of LDL-C, which is a well-known risk factor for atherosclerosis, is increased in SH, the patients with SH were further divided into two subgroups by the levels of LDL-C. In summary, these subjects were categorized into four groups: healthy control subjects (*n* = 13), newly control subjects with high level of LDL-C (*n* = 12), newly diagnosed SH with normal level of LDL-C (*n* = 11), and newly diagnosed SH with high level of LDL-C (*n* = 15). The definition of SH was an elevated TSH (>4.2 mIU/L) with normal levels of free thyroxine (FT4) (12–22 pmol/ml) and free triiodothyronine (FT3) (3.1–6.8 pmol/ml). The concentration of LDL-C higher than 3.36 mmol/L was defined as the high level, and the value of 0.5–3.36 mmol/L was regarded as the normal level.

### 2.2. Anthropometric Measurements

Body weight (kg) and height (cm) were measured without shoes and/or hats. The BMI was calculated as the weight (kg) divided by the height (*m*) squared. The blood pressure of their right arm was measured three times with a desk-model sphygmomanometer. The systolic blood pressure (SBP) and diastolic blood pressure (DBP) were both determined by the mean value of three measurements.

### 2.3. Sample Collection

After overnight fasting, venous blood samples were drawn and centrifuged for 20 min at 3000 *rpm*. The aliquots of serum samples were separated and stored at −80°C (Thermo Fischer Scientific, USA) for further analysis.

### 2.4. Thyroid Profiles

Thyroid profiles were assessed by measuring the levels of serum TSH, FT3, and FT4 using the chemiluminescent immunometric assay (Elecsys 2010, Roche, Basel, Switzerland). All of the intra-assay and interassay coefficients of variation were less than 5%. The reference ranges for FT3, FT4, and TSH were 3.1–6.8 pmol/ml, 12–22 pmol/ml, and 0.27–4.2 mIU/L, respectively. All the experiments were performed at the clinical laboratory of Provincial Hospital.

### 2.5. Lipid Profiles and FBG

Concentrations of serum fasting blood glucose (FBG), total cholesterol (TC), LDL-C, HDL-C, and triglycerides (TG) were measured using standard enzymatic methods by automated spectrophotometry on the Olympus AU5400 system (Olympus Corporation, Tokyo, Japan). The intra-assay and interassay coefficients of variation were less than 5% and 10%, respectively. The reference ranges for FBG, TC, LDL-C, HDL-C, and TG were 3.9–6.3 mmol/L, 3.6–6.2 mmol/L, 0.5–3.36 mmol/L, 0.8–1.5 mmol/L, and 0.4–1.8 mmol/L, respectively. All the above parameters were obtained from the clinical laboratory of Provincial Hospital affiliated to Shandong University.

### 2.6. Assay for Oxidative Stress Parameter gp91^phox^

The levels of serum soluble gp91^phox^ were determined by the commercially available ELISA kits (Blue Gene Biotech, China) according to the manufacturer's instructions. 100 *μ*L of sample was added into the antibody-coated well, and then, 50μL of conjugated solution was added into each well. After mixture and subsequent incubation for 60 minutes at 37°C, the samples were washed 5 times with washing buffer. 50 *μ*L of enzyme substrates A and B was then added before the reaction was stopped with 50 *μ*L of stop solution. The absorbance was read at 450 nm using a microplate reader immediately (Multiscan MS). The values were expressed in the form of ng/mL. The intra-assay and interassay coefficients of variation were 5.6% and 7.9%, respectively.

### 2.7. Statistical Analysis

The continuous variables were shown by the mean ± standard deviation (SD). The normal distribution was assessed by the Kolmogorov–Smirnov test. For analyzing those continuous variables, the comparisons among the four groups were examined with the parametric one-way ANOVA test followed by a least significant difference (LSD) post hoc test when the normal distribution was conformed. Otherwise, the nonparametric one-way ANOVA Kruskal–Wallis test along with pairwise comparison was applied if a skewed distribution was met. For categorical variables, a chi-squared or Fisher's exact test was selected. The correlations between the variables and gp91^phox^ were assessed by the Spearman and partial correlation analysis. The main effect analysis and interactive analysis were performed with a univariate general linear regression analysis. All the statistical analyses were performed using the SPSS software version 17 (SPSS Inc, Chicago, IL, USA) for Windows. A two-sided *p* valve less than 0.05 was considered statistically significant.

## 3. Results

### 3.1. Clinical Characteristics of the Subjects

**Figure 1:**
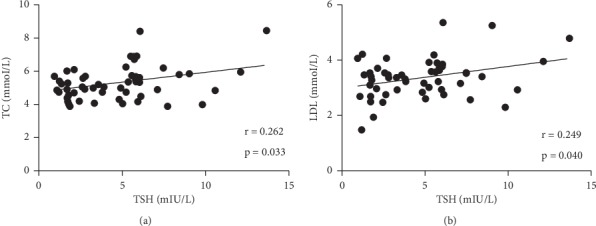
Spearman correlation analysis of TSH with (a) TC and (b) LDL-C. Spearman correlation analysis revealed that the levels of TSH were positively correlated with those of LDL-C (*r* = 0.249, *p*=0.040) and TC (*r* = 0.262, *p*=0.033) in the whole population of 51 subjects. TC : total cholesterol; LDL-C : low-density lipoprotein cholesterol; TSH : thyroid stimulating hormone.

As shown in Table 1, demographic characteristics of gender, age, and BMI were matched among the four groups. The detailed information of lipid profiles (LDL-C, HDL-C, TC, and TG), FBG, and thyroid profiles (FT3, FT4, and TSH) of the four groups is also listed in Table 1. A Spearman correlation analysis revealed that the levels of TSH were positively correlated with those of LDL-C (*r* = 0.249, *p*=0.040) and TC (*r* = 0.262, *p*=0.033) in the whole population of 51 subjects (Figure 1).

### 3.2. gp91^phox^ Levels among the Four Groups

**Figure 2:**
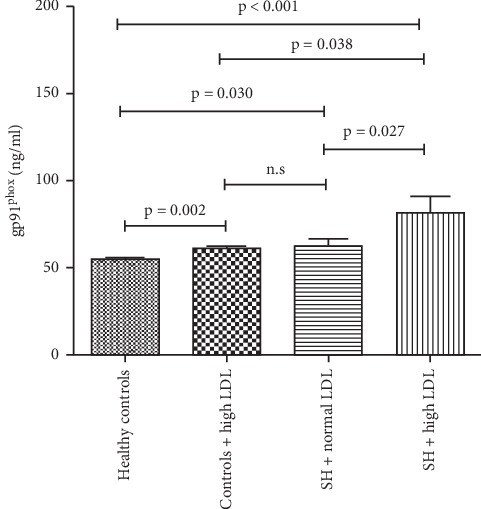
Levels of serum soluble gp91^phox^ of four groups in the study. The levels of serum soluble gp91^phox^ of four groups were compared, respectively. ns, not significant. LDL-C : low-density lipoprotein cholesterol; TH : subclinical hypothyroidism.

The levels of serum soluble gp91^phox^ evidently increased in the patients with SH with high level of LDL-C as compared to the healthy controls (81.52 ± 37.00ug/mL vs. 54.98 ± 1.83ug/mL, *p* < 0.001), controls with high level of LDL-C (81.52 ± 37.00ug/mL vs. 61.21 ± 4.48ug/mL, *p*=0.038), and SH with normal level of LDL-C (81.52 ± 37.00ug/mL vs. 62.82 ± 11.67ug/mL, *p*=0.027), indicating that the level of LDL-C or TSH might exert an effect on that of gp91^phox^. There was also a significant difference between the healthy controls and controls with high level of LDL-C (54.98 ± 1.83ug/mL vs. 61.21 ± 4.48ug/mL, *p*=0.002) and between the healthy controls and SH with normal level of LDL-C (54.98 ± 1.83ug/mL vs. 62.82 ± 11.67ug/mL, *p*=0.030). However, the levels of gp91^phox^ were comparable between the controls with high level of LDL-C and SH with normal level of LDL-C (61.21 ± 4.48ug/mL vs. 62.82 ± 11.67ug/mL, *p*=0.392) (Figure 2).

### 3.3. Correlation Analyses of gp91^phox^ with TSH and LDL-C

**Figure 3:**
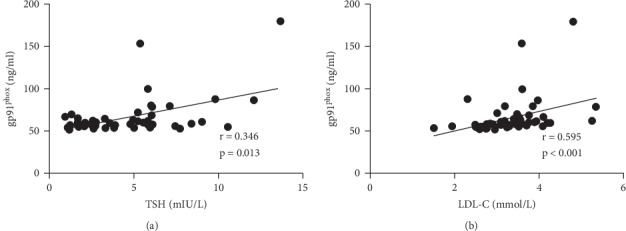
Spearman correlation of TSH and LDL-C with gp91^phox^. LDL-C : low-density lipoprotein cholesterol; TSH: thyroid stimulating hormone.

The levels of gp91phox showed a significant correlation with both the levels of LDL-C (*r* = 0.595, *p* < 0.001) and TSH (*r* = 0.346, *p*=0.013), shown by the Spearman correlation analyses (Tables 2 and 3, Figure 3). Further partial correlation analyses showed that the correlations remained significant with the levels of TSH (when the effect of LDL-C was controlled, *r* = 0.453, *p*=0.001) and LDL-C (when the effect of TSH was controlled, *r* = 0.291, *p*=0.040) even when the effect of another factor was controlled (Table 3).

The main effect analysis by using a univariate general linear regression model also showed an independent main effect of either the levels of LDL-C (*p*=0.041) or TSH (*p*=0.022) on the levels of gp91^phox^. Besides, the interactive analysis demonstrated no interaction between the levels of LDL-C and TSH (*p*=0.299) in terms of the levels of gp91^phox^, suggesting that LDL-C and TSH were both independent predictors of gp91^phox^.

## 4. Discussion

Our study first demonstrated the elevated levels of gp91^phox^ in SH and it advocated that SH is tightly correlated with the increased oxidative stress and risk of atherosclerosis [4, 11]. NOX family is a group of key enzymes of the innate immune system in various cell lines [24–26]. Several NOX homologs, namely, NOX1, NOX3, NOX4, and NOX5, which are relevant to the production of oxidant species, have been recently detected in the cardiovascular system [27]. One experimental study using blood cells demonstrated that 90% of serum soluble gp91^phox^ was derived from the platelets, neutrophils, and monocytes [20]. Besides, NOX-derived superoxide anions are an effective scavenger of NO and could mediate LDL-C oxidation in atherosclerosis [25, 26, 28]. And monocytes/macrophages and platelets in the vascular walls are also one of the most significant sources of these anions. In this study, our results might shed light on a novel predictive biomarker as well as therapeutic target for measuring the oxidative stress in SH.

The influence of SH on the lipid metabolism is another debated issue. Consistent with several previous studies, our results showed the increased levels of TC and LDL-C in SH [2, 29, 30]. Similarly, our study pointed towards a positive correlation between the levels of TSH and TC and LDL-C [7, 31, 32]. One recent research also indicated a potential mechanism for hypercholesterolemia involving a direct action of TSH on the liver [33].

Accumulating evidence supported an enhanced intensity of oxidative stress in hypercholesterolemia. Several experimental and clinical studies have also shown that hypercholesterolemia is associated with the enhanced production of ROS in the endothelial cells and platelets [28, 34]. Pignatelli et al. demonstrated that the levels of gp91^phox^ were significantly higher in the subjects with hypercholesterolemia [20]. In our study, an increased level of gp91^phox^ was noted in the patients with SH and high level of LDL-C. And we further observed a positive correlation of the levels of LDL-C and gp91^phox^ by the Spearman correlation analysis and partial correlation analysis, as well as an independent predictive role of LDL-C in the levels of gp91^phox^, by the main effect analysis and interactive analysis.

The excess TSH per se might also account for the increase in oxidative processes in SH. It is widely accepted that the endothelial dysfunction is an early step in the development of atherosclerosis and is associated with an increased risk of cardiovascular events [35]. The endothelium-dependent vasodilation impairment is characterized by the decreased nitric oxide (NO) availability and, frequently, by the increased oxidative stress [36, 37]. Recent studies have validated that TSH is able to induce IL-6 and TNF-*α* secretion in vitro [38, 39], which might be involved in the modulation of vascular function by increasing NO metabolites in vivo [40]. It suggests a possible link between TSH and inflammation and oxidative stress. In a recent study, the enhanced inflammation and oxidative stress have been demonstrated in the patients with differentiated thyroid carcinoma treated with high concentrations of recombinant human TSH [22]. Our study showed that the level of TSH was another independent predictive indicator of the changes in the levels of gp91^phox^, and it also significantly and positively correlated with the levels of gp91^phox^. This indicates that both hyperthyrotropinemia and dyslipidemia might indeed play an independent role in the elevation of oxidative stress level in SH. In summary, the measurement of serum gp91^phox^ could be applied as a useful and promising biomarker and predictor to evaluate the levels of oxidative stress as well as the clinical prognosis in patients with SH.

Several limitations existed which should be paid to interpret the results in this study. First, the observational study design inevitably introduced a source of potential bias. Second, this cross-sectional study mainly reported descriptive data and does not allow to evaluate the causal relationships between the gp91^phox^ increase and thyroid function evolution. Third, the simple sizes of the whole cohort and each subgroup were relatively small and it was a single-center study. Fourth, the level of gp91^phox^ might be affected by other potential confounding variables.

## 5. Conclusions

In conclusion, our study showed an increase in the levels of gp91^phox^, a novel biomarker for evaluating the oxidative stress, in patients with SH. And the levels of LDL-C and TSH might both possess independent effects on the levels of gp91^phox^. Future multicenter prospective studies with larger cohorts are warranted to evaluate those results.

## Figures and Tables

**Table 1:** Clinical characteristics of four groups in the study.

	Healthy controls	Controls with high LDL	SH with normal LDL	SH with high LDL	*p*1^*∗*^	*p*2^#^
	*n* = 13	*n* = 12	*n* = 11	*n* = 15		
Gender (F/M）	10/3	9/3	9/2	11/3	0.978	-
Age (years)	50.38 ± 3.52	50.33 ± 4.12	54.09 ± 6.88	51.60 ± 7.60	0.202	0.423
BMI (kg/m^2^)	22.9 ± 2.21	23.15 ± 2.48	24.48 ± 2.62	24.44 ± 2.31	0.343	0.172
TC (mmol/L)	4.56 ± 0.37	5.41 ± 0.39	5.54 ± 0.53	6.31 ± 1.00	<0.001	<0.001
LDL-C (mmol/L)	2.27 ± 0.53	3.65 ± 0.30	2.86 ± 0.28	4.02 ± 0.61	N/A	N/A
HDL-C (mmol/L)	1.35 ± 0.13	1.19 ± 0.13	1.18 ± 0.16	1.45 ± 0.34	0.021	0.782
TG (mmol/L)	1.05 ± 0.34	1.16 ± 0.36	1.15 ± 0.34	1.40 ± 0.34	0.144	0.020
FBG (mmol/L)	5.08 ± 0.41	4.97 ± 0.65	5.50 ± 0.73	6.58 ± 2.18	0.008	0.169
FT3 (pmol/L)	5.20 ± 0.62	5.47 ± 0.36	4.93 ± 0.71	4.73 ± 0.28	0.002	0.028
FT4 (pmol/L)	16.41 ± 1.58	17.76 ± 1.65	15.36 ± 1.85	15.59 ± 2.04	0.013	0.397
TSH (mIU/L)	2.22 ± 0.92	2.19 ± 0.85	6.62 ± 1.98	7.17 ± 2.58	N/A	N/A

^*∗*^, nonparametric one-way Kruskal–Wallis for all the four groups; ^*#*^, healthy controls vs. SH with high LDL. BMI : body mass index; TC : total cholesterol; LDL-C : low-density lipoprotein cholesterol; HDL-C : high-density lipoprotein cholesterol; TG : triglyceride; FBG : fasting blood glucose; FT3 : free triiodothyronine; FT4 : free thyroxine; TSH: thyroid stimulating hormone.

**Table 2:** Spearman correlation of different variables with gp91^phox^.

Variables	*r*	*p*
Age (years)	−0.120	0.400
BMI (kg/m^2^)	0.27	0.314
HDL (mmol/L)	−0.004	0.978
LDL (mmol/L)	0.595	<0.001
TC (mmol/L)	0.547	<0.001
FT3 (pmol/L)	−0.105	0.465
FT4 (pmol/L)	0.026	0.854
TSH (mIU/L)	0.346	0.013
TG (mmol/L)	0.173	0.225
FBG (mmol/L)	0.156	0.274

BMI : body mass index; TC : total cholesterol; LDL-C : low-density lipoprotein cholesterol; HDL-C : high-density lipoprotein cholesterol; TG : triglyceride; FBG : fasting blood glucose; FT3 : free triiodothyronine; FT4 : free thyroxine; TSH: thyroid stimulating hormone.

**Table 3:** Correlation analyses between gp91^phox^ with TSH and LDL-C.

	Correlation coefficient	Controlling factor	*p*
TSH	0.494	—	<0.001
TSH	0.453	LDL-C	0.001
LDL-C	0.359	—	0.010
LDL-C	0.291	TSH	0.040

Spearman correlation coefficients and partial correlation coefficients of TSH and LDL-C with gp91phox were calculated. BMI : body mass index; TC : total cholesterol; LDL-C : low-density lipoprotein cholesterol; HDL-C : high-density lipoprotein cholesterol; TG : triglyceride; FBG : fasting blood glucose; FT3 : free triiodothyronine; FT4 : free thyroxine; TSH: thyroid stimulating hormone.

## Data Availability

All data generated or analyzed during this study were included in this article.
